# Life-Stage–Dependent Variation in Gastrointestinal Microbial Communities of *Drosophila melanogaster*

**DOI:** 10.1007/s00248-026-02757-8

**Published:** 2026-04-10

**Authors:** Arnau Rocabert, Laia Pareras, Raquel Egea, Mohamed Alaraby, Laura Rubio, Ricard Marcos, Alba García-Rodríguez, Alba Hernández

**Affiliations:** 1 Group of Mutagenesis, Department of Genetics and Microbiology, Faculty of Biosciences, Universitat Autònoma de Barcelona, Campus of Bellaterra, Cerdanyola del Vallès, Barcelona 08193 Spain; 2 Zoology Department, Faculty of Science, Sohag University, Sohag, 82524 Egypt

**Keywords:** *Drosophila*, Microbiota, Age-related effects, MinION nanopore sequencing, Age-associated dysbiosis

## Abstract

*Drosophila melanogaster* has been a useful biological model since its first use, more than 100 years ago. Mainly due to its genetic similarity with humans, as it allowed it to be an accurate representation of how different biological systems work, such as immune function, illness related pathways, gene inheritance, egg development and, in this case, the microbial ecosystem. Age-related changes in the gut microbiota of *D. melanogaster* offer valuable insights into the dynamic relationship between a host and its microorganisms across lifespan and can be extrapolated to other organisms. This study focusses on the potential changes that microbiota undergoes across the several stages of the fly life cycle, generating robust and high-resolution data using MinION nanopore sequencing as a cutting-edge approach to microbiota analysis Our findings highlight the highly dynamic nature of the gut microbiota across the *D. melanogaster* lifespan and underscore the necessity of explicitly accounting for developmental stage and chronological age in microbiota-focused studies. Moreover, a clearer understanding of these temporal microbial shifts provides valuable insight into how host-microbiota interactions are shaped during development, maintained during adulthood, and ultimately altered during aging. Currently, the gut microbiota stabilized following post-eclosion establishment, while age-associated dysbiosis and immune decline have not yet emerged. Consequently, using flies around 1-week-old maximizes experimental consistency and sensitivity, making them an optimal model for investigating microbiota-targeted interventions.

**Supplementary Information:**

The online version contains supplementary material available at 10.1007/s00248-026-02757-8.

## Introduction

Since the early 20th century, *Drosophila melanogaster*, commonly known as the fruit fly, has stood as one of the most important model organisms in biological research. Its short generation time, well-characterized genetics, and the availability of powerful molecular tools have made *D. melanogaster* a cornerstone in diverse fields ranging from developmental biology and neuroscience to genetics and aging [1]. The fly’s relatively simple yet evolutionarily conserved physiology has enabled scientists to explore fundamental biological processes that are often mirrored in higher organisms, including humans [2]. In recent years, *D. melanogaster* has emerged as a valuable system for studying host-microbe interactions, particularly in the context of the gut microbiota [3]. Although the fly harbors a far less complex microbiota than mammals, this simplicity is an advantage for mechanistic studies. The *D. melanogaster* gut provides a tractable and genetically manipulable model to dissect the molecular dialogues between host and microbes, allowing researchers to explore how microbial communities influence host physiology, immunity, metabolism, and behavior [4].

Parallel to advances in model organism research, our understanding of human microbiota has expanded dramatically. The human body is colonized by trillions of microorganisms, primarily bacteria, which reside in niches such as the skin, oral cavity, and most densely, the gastrointestinal tract [5]. This complex ecosystem plays a crucial role in human health, contributing to nutrient absorption, vitamin synthesis, immune regulation, and protection against pathogens. Disruptions in the composition or function of the gut microbiota, known as dysbiosis, have been linked to a wide range of diseases, including inflammatory bowel disease, obesity, diabetes, neurodegenerative disorders, and even cancer [6].

One critical factor that shapes the composition and function of the microbiota is aging. As humans age, the gut microbiota undergoes dynamic changes, often characterized by a decrease in microbial diversity and a shift toward pro-inflammatory profiles [7]. These age-related microbial shifts have already been published regarding mice [8] and zebrafish [9] and are thought to contribute to the phenomenon of “inflammaging”, a chronic, low-grade inflammation associated with aging and may play a role in age-related declines in immune function, metabolic regulation, and tissue homeostasis [10]. Notably, studies in both humans and animal models have shown that microbiota composition can influence the rate and quality of aging, suggesting that the gut microbiota is not merely a passive passenger but an active participant in the aging process [11]. Even though aging is not reversible, the act of maintaining a healthy and balanced microbiota can help prevent diseases in old age [12]. Model organisms like *D. melanogaster* provide an ideal platform to investigate the causal relationships between microbial communities and aging. The fly’s short lifespan and the ease of manipulating both host and microbial components make it a powerful tool for exploring how age-associated changes in microbiota influence health span and longevity [13]. Moreover, cross-genera comparisons enable the identification of conserved pathways and mechanisms through which microbiota contributes to aging, with implications for understanding human aging and developing microbiota-targeted therapies.

While *D. melanogaster* has long been a cornerstone model organism in genetics, there remains no clear consensus among researchers regarding the optimal age of flies for experimental treatments. A general trend suggests that younger adults are often preferred, but different examples illustrate the lack of standardization in experimental design concerning fly age, particularly in studies involving the gut microbiota of *D. melanogaster*. To solve this point, and as an initial step, a systematic review of fifty publications concerning experimental research on the microbiota of *D. melanogaster* was undertaken to determine which has been the most adopted starting age for microbiota-related treatments. As illustrated in Supplementary Fig. 1S and associated Table 1S, the literature reveals no clear consensus regarding the appropriate developmental stage at which to begin such interventions. Although a modest tendency can be observed towards the use of flies in approximately 1-week post-eclosion, the variability across studies is substantial. Some experiments employ individuals as young as 8-hours-old, whereas others make use of flies up to one month of age. This broad divergence in methodological practice highlights a significant lack of standardization, which may complicate cross-study comparisons and hinder the establishment of robust, reproducible conclusions within the field.

Considering this lack of standardization, the present study aims to provide a clearer and more systematic characterization of age-associated differences in the *D. melanogaster* gut microbiota across the life cycle, while also generating robust and high-resolution data using MinION nanopore sequencing as a cutting-edge approach to microbiota analysis [14]. By combining age-stratified sampling in key points of the fly life cycle usually used in studies (larvae stage, post-eclosion, young adults and mid-life adults with 16 S rRNA gene sequencing of DNA extracted from the fly gut, this work seeks to elucidate how host age influences microbial composition and abundance, with the broader objective of establishing optimal and reproducible conditions for microbiota-focused studies in this model organism. Importantly, despite the growing adoption of nanopore technologies in microbiota research, their application to *D. melanogaster* gut microbiota remains limited, with relatively few studies leveraging the long-read capabilities and real-time sequencing advantages of this platform. As a result, much of the existing literature continues to rely on short-read or earlier-generation sequencing approaches. By addressing this methodological gap, our findings contribute to the development of more standardized, age-based criteria for experimental design in *D. melanogaster* microbiota research and provide novel, reliable insights into an underexplored area of the field.

## Materials and Methods

### *Drosophila* Culturing

*D. melanogaster* of the Canton-S strain was selected as the model organism for the study. Flies were kept at 25 °C with a light/dark cycle of 12 h each, in 130 mL crystal flasks containing a combination of fresh yeast (120 g in 500 c.c. of water), corn (170 g in 500 c.c. of water), agar (10 g), salt (2 g), nipagin (2.5 g diluted in 20 c.c. ethanol as a preservative) and propionic acid (2.5 c.c.), forming and adequate medium for their feeding and growth. Their flask also included a piece of paper to provide both a resting place as well as an adequate place for flies to lay their eggs. This paper was also coated with the antifungal agent Tedion (250 mL of Tretadifon pestanal in 2.5 L of acetone). This antifungal has no documented effect on microbiota. All flies were transferred to a new clean and sterile flask every 4 days; to avoid the fusion of different generations, each flask housed around 200 flies, 100 male and 100 female.

The selected time points in the life cycles of *D. melanogaster* were selected taking into account some of the most common ages at which different researchers use flies and they were as follows: (1) Stage 3 larvae were selected (5 days after egg eclosion), as they are in the larval stage where they are bigger, consume more food, and thus have a more active and changing microbiota. Then, juvenile flies were selected in two different groups, (2) after emerging from pupa (4-day-old pupa were monitored every 12 h and flies selected as soon as they hatched) as the second group (1-day-old flies), and (3) 1-week-old flies as the third group. Finally, a fourth group ranging from (4) 4-week-old was selected as the oldest group. All these groups represent rather common time points in different experiments from other authors. From this point onwards, treatments consisted of three groups each containing a pool of 50 flies with a 1:1 male-to-female ratio. All different conditions can be seen in Table 1.

**Table 1:** Table depicting the different husbandry conditions of each age group

	Larvae	1-day-old	1-week-old	4-week-old
Age (total days since hatching)	5	9	16	37
Sex	1:1 male/female	1:1 male/female	1:1 male/female	1:1 male/female
Number of pools	3	3	3	3
Individuals	50 per pool	51 per pool	52 per pool	53 per pool
Replicates	3	3	3	3
Husbandry variables	Time in the flask	Time in the flask	Time in the flask	Time in the flask

### *Drosophila* Gut Extraction

Dissection was the chosen method to extract the flies’ guts. For the larval stage, a surface sterilization wash was performed using one wash of 10% of sodium hypochlorite solution and three rinses in sterile 1X PBS for 10 s each. After this cleaning, larvae were picked with sterile tweezers and put in a Petri dish. Both the head and the rear of the larvae were squeezed, and then the gut was extracted by pulling from the back end of the larvae. For adult flies, both groups were dissected with the same method. First, the flies were anaesthetized and washed with ethanol for 10 s followed by a rinse with PBS for another 10 s. Then, using sterile tweezers in a Petri dish, the thorax was held in place with the second tweezers pulled from the abdomen, separating both parts and exposing the gut, the midgut was then collected. In all cases, the guts were pooled according to replicates and placed in a 1.5 mL Eppendorf tube and froze at −80 °C until later use. All these procedures were performed in sterile conditions in a laminar flow cabinet.

### DNA Extraction

The extraction of DNA from the collected guts was performed using the ZymoBIOMICS™ DNA Miniprep Kit (Zymo Research Corporation, Orange, CA, USA), which allows for accurate DNA extraction and purification from different types of samples, including guts, and has already been used by our group [15]. The quantity of isolated genomic DNA was measured using a fluorometric assay (Qubit 4 Fluorometer, Thermo Fisher Scientific) and stored at −20 °C until further use.

### DNA Preparation and Sequencing Protocol

Once microbial DNA had been isolated from the samples, the first step taken was a PCR of the whole region of the 16 S gene, as such, expected amplicon is around 1.500 bp. The primers used for this procedure were as follows [16]:

*forward primer* (27 F):

5’ TTTCTGTTGGTGCTGATATTGC-[AGAGTTTGATCMTGGCTCAG] 3’.

and *reverse primer* (1492R):

5’ ACTTGCCTGTCGCTCTATCTTC-[CGGTTACCTTGTTACGACTT] 3’.

DNA amplification was carried out using a BIOER XP thermal cycler. The mix consisted of 12.5 µL of LongAmp^®^ Hot Start Taq 2X Master Mix (New England Biolabs), 15 ng of DNA, 0.4 µM of each primer, and water up to 25 µL. The different cycles in the thermal cyclers were an initial denaturation at 95 °C for 3 min, followed by 5 cycles of amplification (95 °C for 15 s, 55 °C for 15 s, and 65 °C for 90 s). This was followed by 30 additional cycles (95 °C for 15 s, 62 °C for 15 s, and 65 °C for 90 s), and a final elongation at 65 °C for 2 min. After this procedure, a purification step was performed to remove all the different reagents from the PCR that could have a negative effect on subsequent reactions. For this, the AMPure XP bead system (Beckman Coulter, Inc.) was used according to the manufacturer’s instructions. Finally, the DNA was quantified as mentioned before.

After the 16 S PCR, a barcoding PCR was performed to give each sample an individual tag that will allow them to identify later. For that, a mixture is used consisting of 25 µL of LongAmp^®^ Taq 2X Master Mix (New England Biolabs), 154 fmol (150 µg) of the previously purified PCR product, 1 µL of the desired barcode for each sample from the EXP-PBC096 barcoding kit (Oxford Nanopore Technologies, ONT), and Milli-Q water to bring the total volume to 50 µL. The temperature profile for this PCR was as follows: an initial denaturation at 95 °C for 3 min, followed by 15 cycles (95 °C for 15 s, 62 °C for 15 s, and 65 °C for 90 s), and a final elongation step at 65 °C for 2 min. After the amplification, the PCR product was purified using the AMPure XP bead system (Beckman Coulter, Inc.), with a bead-to-sample volume ratio of 0.6:1, this ratio selects for DNA lengths around 1.5 kb, as our expected amplicon, thus avoiding artifacts and chimeras. Agarose gel electrophoresis was performed to assess the presence and integrity of DNA in selected samples, along with a blank control, as shown in Figure S1.

Once barcoded, all the samples were pooled into an individual library at a desired ratio to achieve a total DNA mass of 3000 ng in a volume of 147 µL and once again it was quantified with Qubit 4 Fluorometer, and the samples were stored at −20 °C overnight for further use.

Following the last step, a reparation of the DNA was carried out, which also phosphorylates the 5’ ends and adds tails to the 3’ ends of the DNA fragments. This step requires 49 µL of the pooled DNA, as well as 1 µL of a DNA control sample, 7 µL of NEBNext Ultra II End Prep Reaction Buffer (New England Biolabs), and 3 µL of NEBNext Ultra II End Prep Enzyme Mix (New England Biolabs). The thermal cyclers are 20 °C for 5 min, followed by 65 °C for 5 min. As before, the DNA was purified using the AMPure XP bead system (Beckman Coulter, Inc.), with a bead-to-sample volume ratio of 1:1. After the reparation, the DNA needs to have an adapter attached to it, so it can access the nanopores in the MinION flowcell. This was performed by mixing 60 µL of the repaired DNA with reagents from the SQK-LSK114 Ligation Sequencing Kit (ONT). The reaction mixture contained 25 µL of Ligation Buffer, 5 µL of Adapter Mix H, and 10 µL of NEBNext Quick T4 DNA Ligase (New England Biolabs). The mixture was then incubated for 10 min at room temperature, purified using the AMPure XP bead system (Beckman Coulter, Inc.) and quantified by the Qubit 4 Fluorometer (ThermoFisher Scientific). This library is now ready for sequencing and can be stored at 4 °C for short-term use or at −80 °C for long-term storage (over 3 months).

The created library was then loaded in the MinION flowcell and the metagenomic sequencing was carried out using the ONT MinION platform. This process was carried out with 45 fmol of DNA and the priming of the flow cell followed the manufacturer’s instructions (ONT, SQK-LSK114), which involved unloading any residual or storage buffers from the flow cell and refilling it with a loading buffer, along with the prepared DNA sample.

### Bioinformatic Analysis

All 16 S rRNA sequences were obtained by the Min-KNOW suite and base called and demultiplexed with Guppy 3.0 (ONT). Reads were filtered byquality (> Q8) at the default parameter using Dorado v7.3.11 (ONT). Both Guppy and Dorado are programs implemented inside the MinION sequencer. Taxonomic assignments at the genus level and estimation of abundance were carried out with Emu 3.5.1 [17], the default database of Emu is a combination of rrnDB v5.6 and NCBI (National Center for Biotechnology Information) 16 S RefSeq downloaded on 17 September 2020. [18–20]. Afterwards, taxa with single read-counts were removed. In addition, a low count filter was set to a minimum read count of 10. Finally, filtered data was normalized using total sum scaling (TSS). Filters as well as plots and analysis of microbiota structure and diversity were made with MicrobiomeAnalyst [21] after adapting the format with a custom script in Python.

### Statistical Analysis Metrics

#### Alpha Diversity

To specifically estimate genus richness, the Chao1 index [22] was employed. Chao1 is a non-parametric estimator that accounts for unseen genera by incorporating the number of rare taxa (i.e., singletons and doubletons) into its calculation. This makes it particularly useful in microbial ecology studies, where low-abundance genera can contribute significantly to overall community structure but may be underrepresented in sequencing data. The choice of Chao1 was intended to ensure that richness estimates were not skewed by under-sampling or sequencing depth variability. For the statistical analysis of differences in alpha diversity among age groups, the Kruskal-Wallis test was applied [23]. This non-parametric method is suitable for comparing more than two independent groups when the data does not follow a normal distribution, which is often the case with ecological diversity metrics. By using the Kruskal-Wallis test, the analysis avoids assumptions of homoscedasticity and normality, increasing the robustness of the findings. A significant result from this test would suggest that at least one age group differs significantly from the others in terms of microbial richness.

#### Beta Diversity

To evaluate the differences in microbial community similarity and stability between age groups, a PERMANOVA (Permutational Multivariate Analysis of Variance) [24] was performed. PERMANOVA is a non-parametric method that partitions the dissimilarity matrix among sources of variation and tests the significance of group differences using permutation-based inference. This approach does not rely on assumptions of normality, making it particularly appropriate for complex, high-dimensional ecological data such as microbiota profiles. A PERMDISP (permutational analysis of multivariate dispersions) was performed to assess the homogeneity of group dispersions and to determine whether differences observed in the PERMANOVA analysis could be attributed to significant differences in within-group variance. For visualization of the relationships between samples, Principal Coordinates Analysis (PCoA) [25] was employed as the ordination method. PCoA reduces the complexity of the dissimilarity matrix into a lower-dimensional space, typically two or three axes, while preserving the distance relationships between samples as closely as possible. This facilitates the graphical interpretation of patterns in the data. In the resulting PCoA plots, samples that cluster closely together indicate more similar microbial communities, whereas samples that are farther apart reflect greater compositional divergence.

By integrating Bray-Curtis dissimilarity, PERMANOVA, and PCoA, this analysis provides a robust framework for identifying significant shifts in microbiota composition across the different age groups of *D. melanogaster*. These methods collectively enable a deeper understanding of how microbial communities evolve throughout the fly’s lifespan and how stability or variability in microbial composition correlates with developmental and physiological changes.

#### Linear Discriminant Analysis Effect Size (LEfSe)

The final analysis performed was a Linear Discriminant Analysis Effect Size (LEfSe) [26], a robust tool for identifying microbial taxa that differ significantly across experimental groups. LEfSe combines a Kruskal-Wallis rank-sum test to detect taxa with statistically significant differences in abundance, followed by Linear Discriminant Analysis (LDA) to estimate the effect size of each feature. This approach not only identifies significant differences but also ranks taxa by their relevance in distinguishing between groups.

## Results and Discussion

Evidence indicates that in a wide range of species, including humans and classical model organisms like *D. melanogaster*, gut microbiota diversity is closely related with life-history [27, 28]. Since microbiota structure suffers changes during different life-stages, an unsolved question is at which point the microbiota determination or for the induction of dysbiosis occurs. As indicated in Table S1 and Figure S3, published studies use a wide range of testing points. Thus, for instance, Fujita et al. [29] utilized 5-day-old flies, Yu et al. [30] used 8-day-old flies, and Dodge et al. [31] employed 6-day-old flies. However, other studies have opted for markedly different ages, such as Go et al. [32] with 15-day-old flies, García-Lozano et al. [33] with 1-day-old flies, Wang et al. [34] with 8-hour-old flies, and Daisley et al. [35] with 16-day-old flies. Moreover, most publications do not provide a rationale for the selected age of their experimental subjects, leaving considerable room for interpretation and inconsistency across studies. Consequently, careful description of *D. melanogaster* gut microbiota over time is required.

### Effects of Age on the Abundance of Microorganisms

The first biomarker evaluated in this study was a microbial abundance, which was characterized using two complementary parameters to provide both quantitative and compositional insights into the microbiota [36]. The first parameter, Emu-estimated-counts, refers to the total amount of sequencing fragments assigned to an organism in each experimental group. This metric offers a direct estimate of the overall microbial load present in individuals at different life stages, thereby allowing for a comparison of the general density of microbial populations across age groups. The second parameter, relative abundance, accounts for the proportional representation of specific taxa within the total microbial community. Unlike estimated counts, this measure emphasizes the composition and diversity of the microbiota by identifying shifts in the prevalence of key microbial genera. Relative abundance is particularly informative for detecting ecologically significant changes and for identifying taxa that may exert differential functional roles or influence host physiology in an age-dependent manner. Together, these two parameters provide a comprehensive overview of both the quantity and quality of microbial communities across the studied groups.

**Fig. 1:**
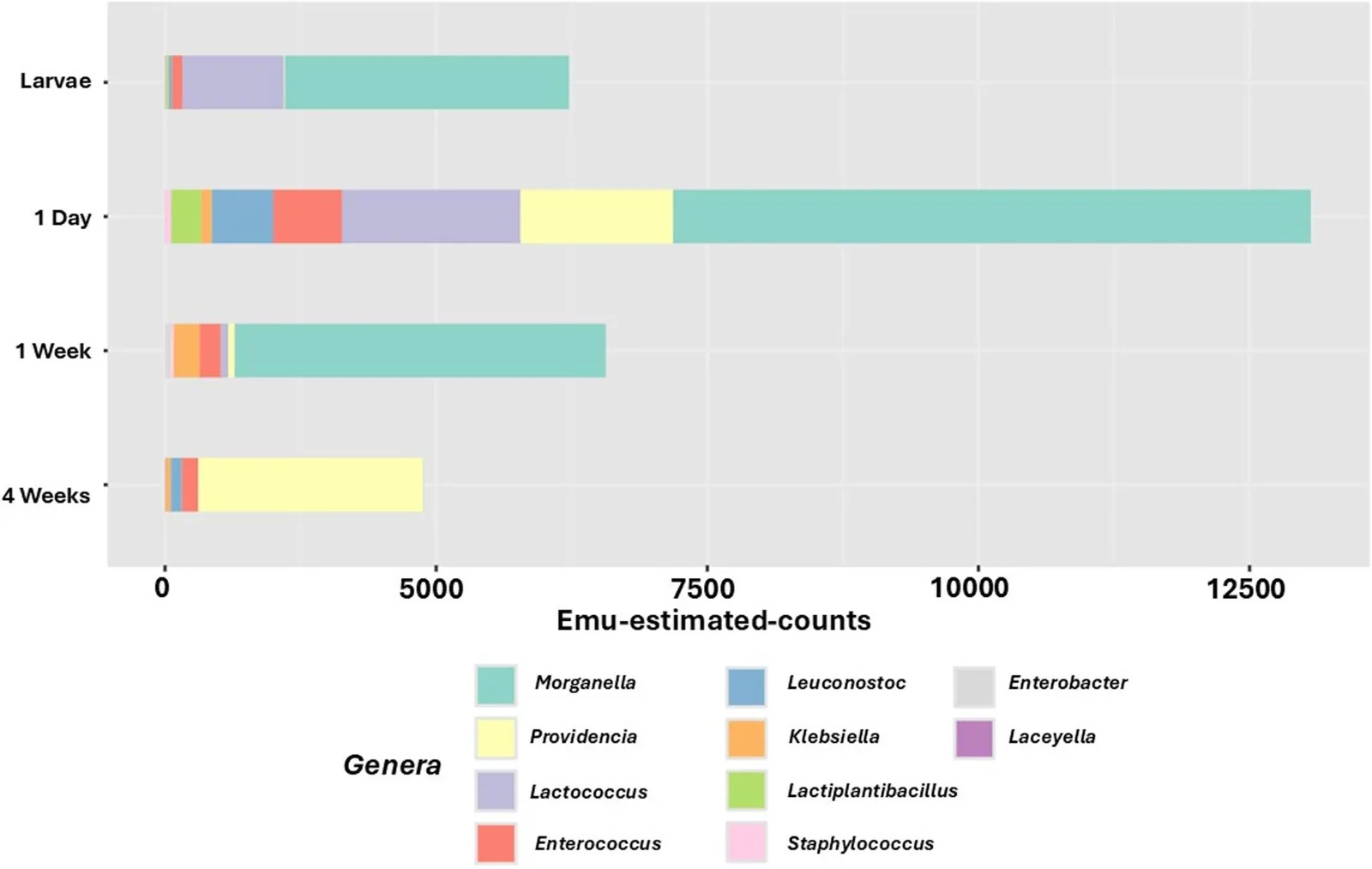
Normalized Emu-estimated-counts. Bar chart depicting counts of top 10 genera across all age groups (each group comprised of 3 replicates of 50 guts pool)

The analysis of microbial counts across developmental stages reveals a dynamic pattern in the total number of counts in each age group. During the larval stage, the estimated counts remain relatively modest, averaging around 6,000 counts. However, upon transition to the first day of adult life, there is a striking increase in counts, with numbers rising to approximately 13,000 counts, more than a twofold increase (Fig. 1). This substantial elevation may be attributed to physiological and anatomical changes occurring during metamorphosis, particularly in the pupal stage. Notably, pupae do not excrete feces, leading to a temporary cessation of gut clearance mechanisms. This creates a closed and relatively secure internal environment in which microbial communities can proliferate without interruption. The absence of gut peristalsis and waste expulsion during this stage likely fosters conditions favorable for bacterial growth and accumulation.

Following eclosion (the emergence of adult flies from the pupa), estimated counts undergo a notable decrease, returning to levels like those observed in the larval phase. Similar reductions have been reported after the reactivation of gut motility and resumption of feeding, digestion, and defecation in the adult fly, as well as the excretion of the older gut from the larval stage [37]. These physiological processes restore the dynamic balance of the intestinal environment, making it less permissive to unchecked microbial proliferation and potentially promoting the re-establishment of a stable microbial equilibrium. These kinds of shifts have also been observed in the metamorphosis steps of other fruit flies [38], but the transitional instability of this group should be considered in experiments and avoided.

As adult flies continue aging, a progressive decline in estimated counts is observed. From 1-week to 4-weeks of age, the total number of counts gradually diminishes. This trend coincides with the onset and progression of senescence, a life stage characterized by a general decline in physiological functions [39]. Several age-related factors may contribute to the observed reduction in microbial counts, including decreased nutrient absorption, changes in gut morphology and permeability, altered immune responses, and potential shifts in the host’s metabolic state. Collectively, these results suggest that microbial colonization may be linked to host developmental and physiological status, with distinct patterns emerging at each life stage. It is important to note that this decrease is not always consistent in literature, as some authors report an increase in microbiota abundance with ageing [40].

Although the extraction procedures inevitably influence the results, incorporating this methodological approach was deemed essential to obtain a clearer and more standardized graphical representation of the effects of the life cycle on the *D. melanogaster* microbiota. Importantly, the impact of microbial loss due to extraction kits has a similar effect across all samples, thereby preserving the validity of comparative analyses. It should be noted that the absence of an internal validation method, such as qPCR possesses a limitation on the extrapolation of this analysis.

**Fig. 2:**
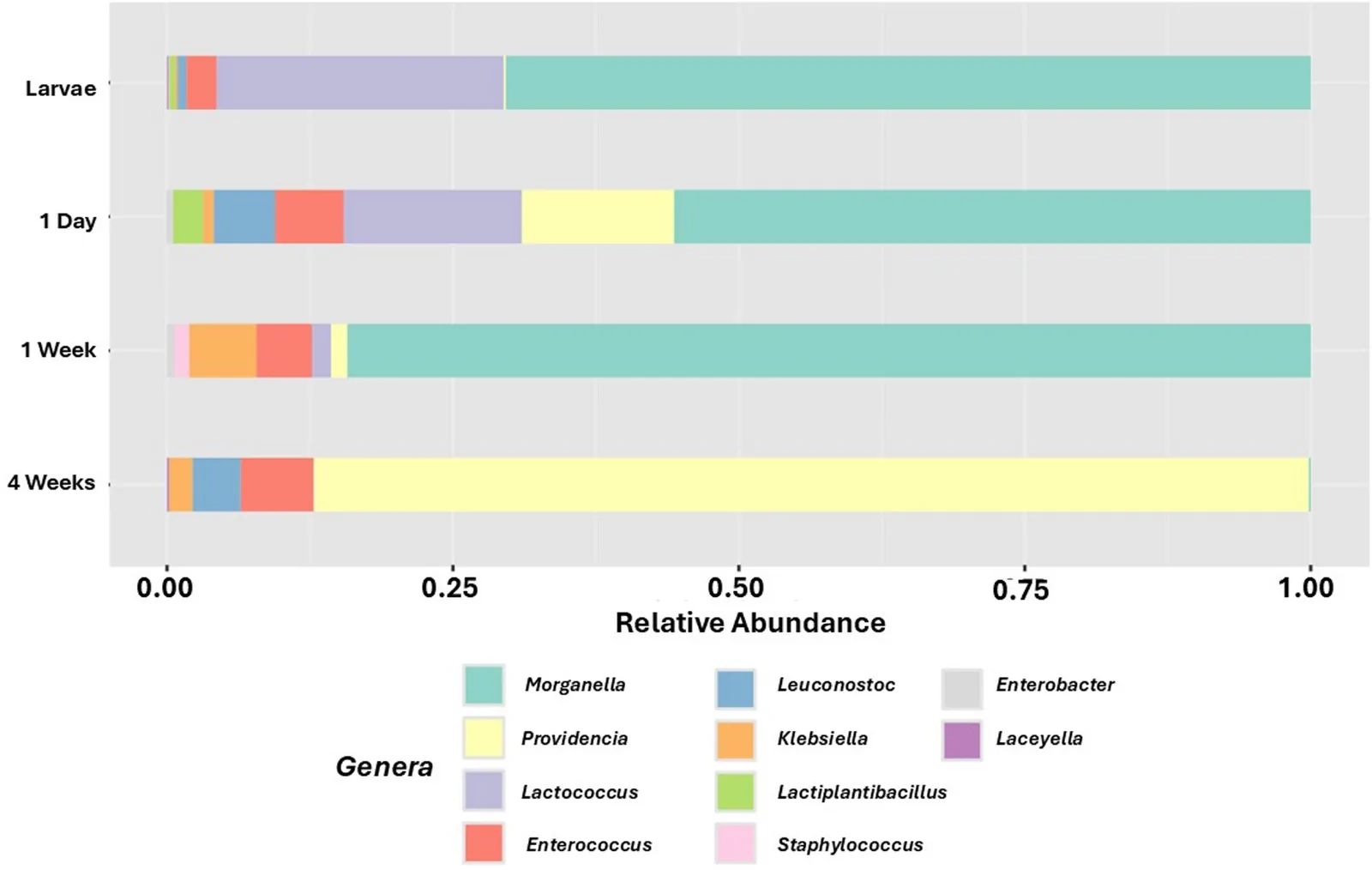
Normalized relative microbial abundance. Bar chart depicting the relative abundances of the top 10 genera across all age groups (each group comprised of 3 replicates of 50 guts pool)

To address the inherent limitations associated with interpreting estimated counts, particularly in the context of technical variability and host size differences, relative abundance analyses were also performed. This complementary approach allowed for the identification and comparison of the most dominant bacterial taxa across experimental groups, providing additional insight into the compositional shifts within the microbial community while also giving more insight to the estimated counts referenced before (Fig. 2).

The results reveal several key findings regarding the composition and dynamics of the *D. melanogaster* microbiota across different life stages. Notably, the dominant microbial genera throughout most of the fly’s lifespan is *Morganella*, which maintains a high relative abundance in larvae, newly emerged adults, and 1-week-old flies. However, this dominance is lost in 4-week-old individuals, where *Morganella* is largely replaced by *Providencia* (Fig. 2). *Morganella*, a genus comprising a single species, *Morganella morganii*, is an opportunistic pathogen commonly associated with a variety of insect hosts, including *D. melanogaster*. Although it is generally considered to have limited pathogenicity in this species, it is a frequent pathogen of laboratory [41]. In contrast, *Providencia* is a well-characterized *D. melanogaster* pathogen genus, that comprises five different species [42], frequently used in experimental models of host-microbe interaction due to its consistent virulence and predictable immune response in flies. These species also exhibit varying levels of pathogenicity and mortality in the fly [43], which were not observed in our treatments. The presence of this second pathogen (*Providencia*) in older flies could be related to the previously mentioned senescence, as when flies grow older not only do their microbiota get affected, but also many other systems such as the immune system, giving a possible explanation to the change, invasion and colonization of a new pathogen in the gut microbiota [43]. This pathogen is also typically found in flies that are grown in laboratories [44].

In addition to these dominant genera, the larval stage, as well as the 1-week-old, are characterized by a notable presence of *Lactococcus*, a commensal bacterium commonly reported as part of the microbiota in *D. melanogaster* [45]. The observed genera distribution patterns suggest dynamic shifts in microbial composition across the life cycle, potentially driven by developmental and physiological changes in the host. In the larvae, the gut environment is highly dynamic due to continuous feeding and defecation, which may lead to rapid turnover and replacement of microbial communities [46]. This constant flux can render larval microbiota more susceptible to environmental influences and transient colonizers as previously mentioned [47]. These compositional shifts in the microbiota may also extend into the pupal stage. Although the pupal gut is functionally inactive, the enclosed and static nature of this developmental stage may allow for the persistence or even selective expansion of certain microbial taxa, potentially influencing the microbial community structure observed in newly emerged adults. These changes, however, seem to only be present some hours after emerging from the pupa stage, as other researchers found that microbiota levels decrease after metamorphosis [35]. In contrast, 4-week-old flies may experience increased microbial instability due to age-related physiological decline, as other researchers explain that senescence is known to impair immune function and gut homeostasis, potentially making the intestinal environment more permissive to opportunistic colonization and pathogenic overgrowth [48]. These findings support the notion that both developmental stage and host aging play significant roles in shaping the structure and stability of the *D. melanogaster* gut microbiota. The second relevant observable characteristic is the presence of different genera, other than the dominant bacteria found. Both in the larval stage and in 1-day-old flies, the group of low abundance bacteria represents a relevant part of the community (30–45%), while older flies have a more dominant community and the other groups seem to represent just a niche (around 15%).

The microbial shifts observed in both the larval stage and the 4-week-old flies (Fig. 2) highlight significant alterations in community structure, with the most pronounced changes occurring in aged individuals. These fluctuations are particularly relevant in the context of experimental research, as they reflect decreased microbial stability in these life stages. Such changes can introduce greater inter-individual variation, complicate the interpretation of results, and potentially reduce experimental reproducibility. Moreover, the increased presence of less common or transient microbial genera in these groups may further confound analyses, especially in studies aiming to assess host-microbiota interactions under controlled conditions. In adult flies, the gut ecosystem may be more homogeneous, as they are predominantly parasitized by a single, more dominant organism. This occurs because *Providencia* successfully can colonize the entire gut, thereby reducing inter-individual variability.

### Microbial Diversity

Another widely utilized biomarker in microbiota research is alpha diversity, which provides an assessment of the within-sample diversity by quantifying the number and distribution of distinct taxa identified in every group. This metric is particularly informative as it reflects both the *richness* (i.e., the total number of unique taxa) and, depending on the index used, and the *evenness* (i.e., how evenly distributed those taxa are). In the present study, alpha diversity was calculated to gain a deeper understanding of how microbial complexity varies across different developmental stages of *D. melanogaster*. Altogether, the use of alpha diversity analysis, particularly via the Chao1 index and Kruskal-Wallis test, provides an essential complementary perspective to abundance-based metrics. It helps characterize not only how many microbial genera are present in each developmental stage, but also how the community complexity evolves over time, offering further insight into the ecological dynamics of the *D. melanogaster* gut microbiota. *P*-value for the overall comparison was not significant (0.2578), *p*-values for pairwise comparisons are as follows: 4-week-old/1-day-old (0.04), 4-week-old/Larvae (0.65), 4-week-old/1-week-old (0.16), 1-day-old/Larvae (0.82), 1-day-old/1-week-old (0.27), Larvae/1-week-old (1).

**Fig. 3:**
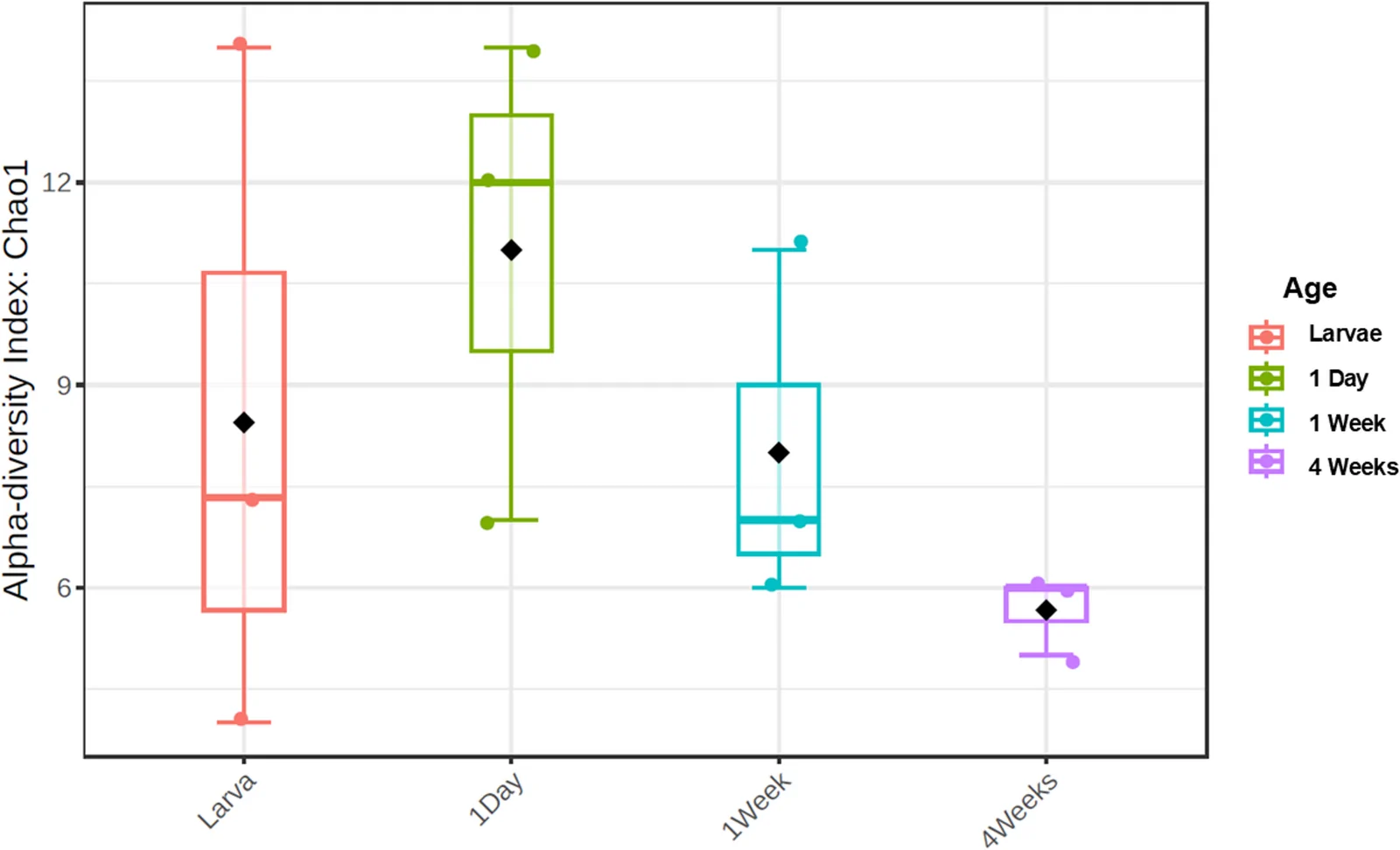
Box plot depicting alpha diversity across all groups after normalization. Colored dots are individual values, black dots are the mean for each group, the rectangle shows the IQR and the colored horizontal line within the rectangle is the median. Significant changes were found between 1-day-old and 4-week-old groups. Chao1 was used as the diversity index and Kruskal-Wallis as the statistical method

Figure 3 shows a clear temporal shift in alpha diversity across the life stages of*D. melanogaster*, highlighting dynamic changes in the complexity of the gut microbiota.

From the larval stage to newly emerged (1-day-old) adult flies, there is a noticeable increase in microbial diversity, suggesting a diversification of the gut microbial community during or immediately after metamorphosis. This increase may be driven by physiological changes in the gut environment, altered feeding behavior, or changes in immune regulation that occur during the transition from larva to adult. This trend is reversed in 1-week-old flies, where a decline in alpha diversity is detected. This reduction may reflect the stabilization or selective pruning of the microbiota following adult emergence, possibly due to the re-establishment of intestinal motility, immune homeostasis, or shifts in dietary intake. The decline becomes even more pronounced in 4-week-old individuals, where microbial diversity drops significantly. This marked decrease in diversity during late adulthood is consistent with the onset of senescence, during which host physiological functions, including immune responses, gut barrier integrity, and metabolic activity, begin to deteriorate. These age-associated changes likely contribute to a less hospitable and more selective environment for microbial colonization, resulting in reduced richness and a more simplified microbial community.

Overall, these findings suggest a clear temporal shift in alpha diversity across the life stages of *D. melanogaster*, although these changes are only significant when comparing to the 4-week-old flies that are already undergoing senescence, they highlight the points already mentioned in previous data representations, stating again that gut microbial diversity in *D. melanogaster* is not static but instead reflects the ongoing interplay between host development, environmental exposure, and physiological aging. The observed patterns reinforce the importance of considering age as a critical factor in microbiota-related studies, as different life stages may harbor distinct microbial communities with potentially different functional consequences and, as such, may have an impact on the results of said studies.

### Community Diversity

Beta diversity is another fundamental metric used in microbiota analysis, providing insight into the differences in microbial community composition between samples or individuals. Unlike alpha diversity, which focuses on the richness and evenness within a single sample, beta diversity quantifies *inter-sample variation*, allowing researchers to assess the degree of similarity or dissimilarity between groups. This metric is particularly useful for evaluating how consistent or heterogeneous microbiota is within each age group, and for detecting global shifts in community structure across developmental stages. In this study, beta diversity was assessed using the Bray-Curtis dissimilarity index [49], an abundance-based measure that captures differences in community composition by considering both shared and unique taxa, as well as their relative abundances. The Bray-Curtis index is well suited for microbiota data due to its sensitivity to ecological gradients and the dominance of specific taxa.

PERMANOVA analysis revealed a strong overall effect, with an R² of 0.8368, an F-value of 13.676, and a p-value of 0.001, indicating that group identity explained a substantial proportion of the variation in beta diversity. Pairwise comparisons did not reach statistical significance after correction (*p* = 0.1). PERMDISP analysis showed no significant differences in within-group dispersion (F = 2.855, *p* = 0.104), indicating that the observed PERMANOVA results are not driven by differences in group variability.

**Fig. 4:**
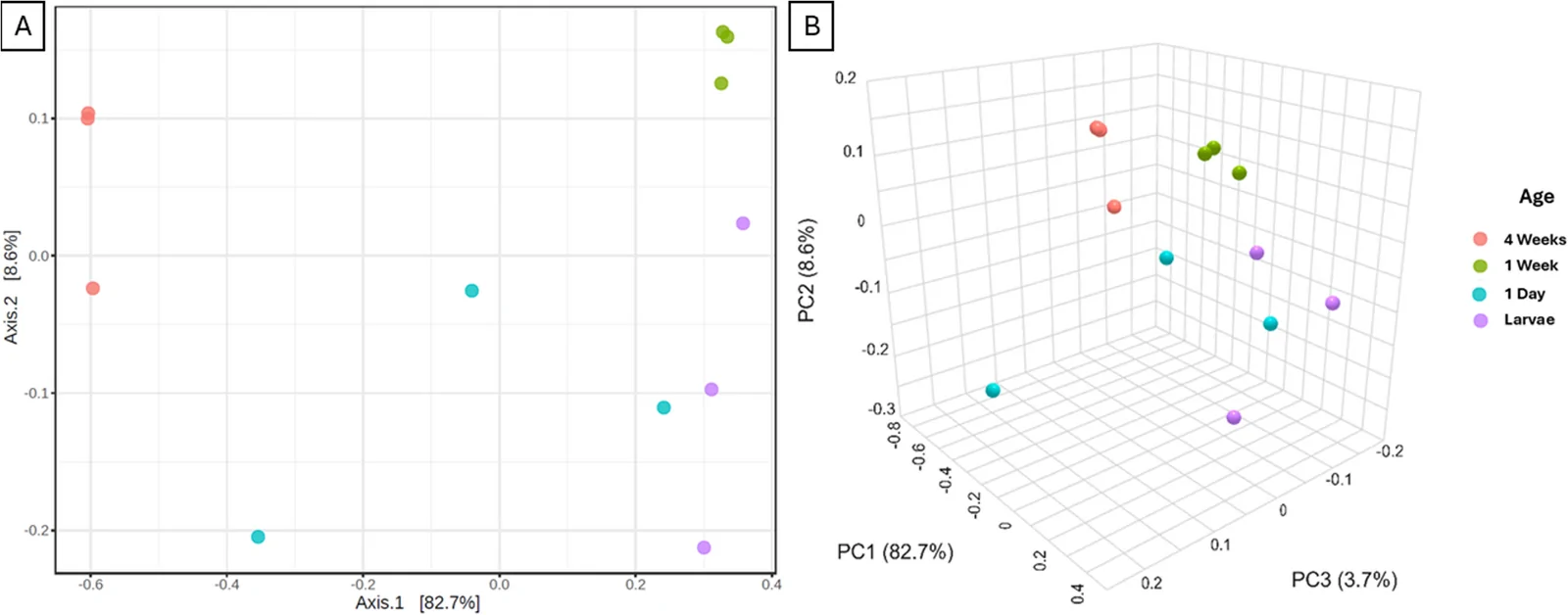
PCoA plot represents beta diversity between all treatments. A depicts PCoA1 and PCoA2 in a two-dimensional plot; B includes PCoA3 in a 3-dimensional plot. Each dot represents one sample. Bray-Curtis as the statistical comparison

As illustrated in Fig. 4, distinct clustering patterns can be observed among samples from different age groups, reflecting notable shifts in gut microbiota composition across the lifespan of *D. melanogaster*.

The PCoA plot displays relatively widespread larval samples, suggesting considerable inter-individual variability. This high dispersion is likely attributable to the physiological and behavioral characteristics of the larval stage, including rapid feeding, high metabolic rates, and continuous excretion [50]. These factors contribute to a dynamic and transient gut environment in which microbial communities are subject to constant turnover and stochastic fluctuations, resulting in less stable and more heterogeneous microbiota profiles.

Upon emergence from the pupal stage, newly eclosed adult flies exhibit even greater dispersion in their microbial communities. This trend may stem from the fact that the pupal stage represents a non-feeding, enclosed phase during which the gut is restructured, and intestinal motility is temporarily halted. The closed nature of the pupal environment may permit extensive proliferation of resident microbes without external perturbation, potentially amplifying the inter-individual differences inherited from the larval stage. These accumulated differences become more apparent once the adult gut becomes functional again, leading to increased variability in early adult microbiota. One last factor that may contribute to this shift is the old larvae’s gut, stored inside adult flies. When *D. melanogaster* emerges from the pupa, this old gut is degraded into the meconium, a waste product that is then expelled [51]. In contrast, 1-week-old adults exhibit the tightest clustering in the PCoA space, indicating relatively stable and homogeneous microbial communities at this stage. This observation supports the hypothesis that, after the initial transition to adulthood, *D. melanogaster* undergoes a period of microbiota stabilization, likely driven by resumed intestinal motility, immune regulation, and consistent feeding patterns. These factors contribute to maintaining microbial homeostasis and reducing inter-individual variability in community composition. Finally, in 4-week-old flies, dispersion increases once again, reflecting a loss of microbial stability during aging. This decline is consistent with the onset of senescence, during which the host’s physiological systems, including immune function, epithelial barrier integrity, and metabolic control, begin to deteriorate. These age-related changes can compromise the host’s ability to regulate its gut microbiota, leading to a resurgence of instability and potentially facilitating colonization by opportunistic or less common taxa.

Altogether, these patterns reinforce the notion that microbial community composition in *D. melanogaster* is influenced by developmental stage and age, with implications for both microbiota-driven host functions and experimental reproducibility. However, our results did not reveal as clearly a pattern as initially anticipated, largely due to the high variability observed in beta diversity among samples. As previously discussed, it is well established that *D. melanogaster* undergoes substantial physiological and microbial changes immediately after emerging from the pupal stage. We hypothesize that this transitional phase, characterized by the expulsion of the larval gut and the onset of adult gut functionality, is a major contributor to the elevated inter-individual variation observed in these early adult samples. To address this issue, future studies will focus on refining the timing of sample collection. Specifically, newly emerged adult flies will be collected only after the complete expulsion of the larval gut has occurred, and normal gut motility has resumed. This adjustment aims to minimize confounding effects linked to post-metamorphic gut restructuring and to improve the reproducibility and interpretability of microbiota data during this critical life stage.

### Linear Discriminant Analysis Effect Size (LEfSe) Analysis

In this study, LEfSe enabled the detection of age-specific microbial biomarkers in *D. melanogaster*, providing insight into how specific taxa characterizes different life stages.

**Fig. 5:**
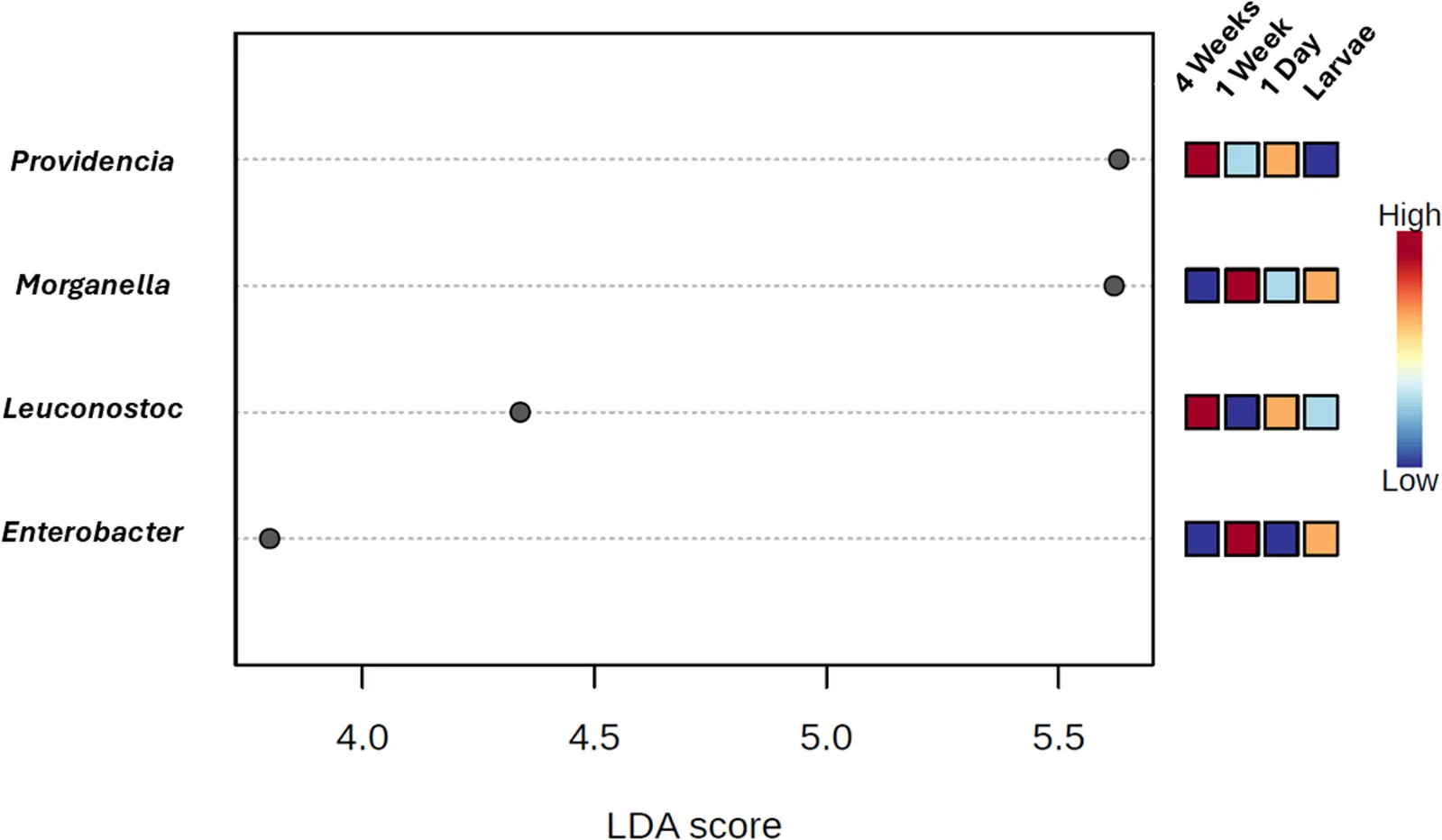
Linear discriminant analysis effect size (LEfSe) graph depicting relevant changes in some genera between age groups. Log LDA score cutoff is 2.0

The LEfSe analysis, depicted in Fig. 5, identified four main bacterial genera exhibiting significant changes in relative abundance across the different age groups of *D. melanogaster*. Two of these genera, *Providencia* and *Morganella*, had already been discussed earlier in the study due to their relevance as known *D. melanogaster* pathogens. *Morganella*, particularly *M. morganii*, was found to be dominant in most age groups except in older flies, where its prevalence declined. In contrast, *Providencia*, specifically *P. rettgeri*, became dominant in 4-week-old flies, consistent with previous reports highlighting its pathogenicity and frequent use as a model for studying host-pathogen interactions in *D. melanogaster* [38].

The third genus, *Leuconostoc*, was also found to be significantly enriched in older flies. Members of this genus have been previously isolated from the *D. melanogaster* gut and are known to exhibit a wide range of interactions with the host. While some *Leuconostoc* species are considered benign commensals with no apparent adverse effects on fly physiology [52, 53], other strains have demonstrated insecticidal properties and may negatively influence host viability under certain conditions [54]. Its increased abundance in senescent flies may reflect age-related changes in gut physiology or immune function that enable colonization by taxa with more variable host interactions.

The final genus, *Enterobacter*, found in 1-week-old flies, is commonly reported in the *D. melanogaster* gut microbiota, and it is generally regarded as beneficial. Several studies have shown that the *Enterobacter* genus can promote host growth, modulate immune responses, and contribute to overall gut homeostasis [55]. However, certain strains within this genus have also been implicated in developmental disturbances and age-related pathologies, suggesting a context-dependent role in host health [56]. The presence of this genus mainly in young flies may be correlated with these kinds of affectations it has on older flies.

Overall, the taxa identified through LEfSe reinforce the idea that aging in *D. melanogaster* is accompanied by pronounced and biologically meaningful shifts in microbiota composition, which may have important implications for host physiology, immunity, and longevity.

## Conclusion

The structure and development of the insect midgut, together with its associated microbial communities, have been the subject of extensive investigation across multiple insect models. Beyond its fundamental roles in digestion and nutrient absorption, the midgut functions as a critical interface mediating interactions between the host and its resident microbiota, thereby influencing host physiology, metabolism, and immune function. In *D. melanogaster*, accumulating evidence indicates that gut microbial composition is closely intertwined with host developmental stage and physiological status [57]. Consistent with this framework, our study demonstrates that the gut microbiota undergoes marked, stage-specific remodeling throughout the *D. melanogaster* life cycle, with two particularly pronounced transition points occurring immediately after post-metamorphic emergence and during late adulthood.

Following emergence from the pupal stage, newly eclosed adult flies exhibited a substantial and reproducible increase in total estimated counts. This transient expansion is likely attributable to the distinctive biological conditions of the pupal phase where feeding is suspended: the gut remains effectively closed to environmental inputs, and intestinal motility is largely absent. Under these circumstances, resident microbial populations may proliferate in the relative absence of mechanical clearance and normal gut turnover. However, this elevated count number is short-lived. As adult flies resume feeding and excretion, shed residual larval gut tissues, and restore normal peristaltic activity the estimated counts rapidly decline to levels comparable to those observed in earlier developmental stages. This reduction likely reflects the reactivation of host-controlled regulatory processes, including immune surveillance, epithelial renewal, and physical clearance mechanisms. Importantly, these developmental transitions were captured not only by changes in overall estimated counts but also by shifts in community structure, as reflected in both alpha and beta diversity metrics. Together, these patterns indicate that host developmental transitions exert coordinated effects on microbial quantity and composition. The concordance of our findings with previous reports in *D. melanogaster* and related tephritid species further supports the notion that metamorphosis constitutes a critical period of microbial instability, followed by a rapid re-establishment of microbial homeostasis in early adulthood [34, 35].

A second major restructuring of the gut microbiota was observed in aged flies, specifically at four weeks of age, a time point associated with the onset of physiological senescence in *D. melanogaster*. At this stage, significant alterations in both microbial composition and total estimated counts were detected, accompanied by increased inter-individual variability. These changes are likely driven by age-associated declines in gut barrier integrity, dysregulation of immune responses, and modifications in feeding behavior and digestive efficiency. As these protective and regulatory mechanisms deteriorate, the host’s capacity to maintain a stable and controlled microbial community appears to diminish, resulting in a more heterogeneous and potentially dysbiotic microbiota. This observation aligns with broader evidence linking aging to microbial instability across animal systems and underscores the importance of considering host age as a major determinant of microbiota composition [58].

Other works have also addressed this idea [59], where the authors report a close interaction between microbiota load and the fly lifespan. Taken together, our findings highlight the highly dynamic nature of the gut microbiota across the *D. melanogaster* lifespan and underscore the necessity of explicitly accounting for developmental stage and chronological age in microbiota-focused studies. Failure to do so may confound experimental outcomes and limit reproducibility across studies. Moreover, a clearer understanding of these temporal microbial shifts provides valuable insight into how host-microbiota interactions are shaped during development, maintained during adulthood, and ultimately altered during aging. Such knowledge is essential for leveraging *D. melanogaster* as a robust model to dissect the mechanistic links between host physiology, microbial ecology, and age-associated decline. Overall, our findings indicate that *D melanogaster* at approximately 1-week-old represents the most appropriate stage for experimental manipulation and treatment-based studies under our laboratory’s husbandry and sequencing conditions, showing lower replicate variability. Currently, the gut microbiota stabilized following post-eclosion establishment, while age-associated dysbiosis and immune decline have not yet emerged. This intermediate state provides a reproducible and physiologically robust baseline, allowing treatment-induced effects on the microbiota to be distinguished from confounding age-related variability. Consequently, using flies around 1-week-old maximizes experimental consistency and sensitivity, making them an optimal model for investigating microbiota-targeted interventions.

## Supplementary Information

Below is the link to the electronic supplementary material.

Supplementary Material 1 (DOCX 130 KB)

## Data Availability

Additional information/data can be made available from the authors upon request.
