# Supplementary material for: Life-Stage–Dependent Variation in Gastrointestinal Microbial Communities of Drosophila melanogaster
Source: Microb Ecol. 2026 Apr 10;89:110. doi: 10.1007/s00248-026-02757-8 (PMC13180772; doi:10.1007/s00248-026-02757-8)
Supplement: Supplementary file 1 [file 248_2026_2757_MOESM1_ESM.docx]

**Studying the gastrointestinal microbial communities along the life cycle of *Drosophila melanogaster***

**Arnau Rocabert^1^, Laia Pareras^1^, Raquel Egea^1^, Mohamed Alaraby^1,2^, Laura Rubio^1^, Ricard Marcos^1^, Alba García-Rodríguez^1,*^, Alba Hernández^1,*^**

*^1^Group of Mutagenesis, Department of Genetics and Microbiology, Faculty of Biosciences, Universitat Autònoma de Barcelona, 08193 Cerdanyola del Vallès, Spain.*

*^2^ Zoology Department, Faculty of Science, Sohag University, Sohag 82524, Egypt.*

**SUPPLEMENTARY MATERIAL**

**Supplementary Figure S1.** Agarose gel electrophoresis of selected samples (lanes 1–6) and a negative control (lane 7). The lanes show DNA fragments of approximately 1.5 kb in length.

**Figure S2:** Read count of different samples as represented in Figure S2.

**Supplementary Figure S3.** Bar chart depicting different papers and their stated age when starting treatments with *Drosophila* (Keywords used in the search:” *Drosophila melanogaster*”, “Microbiota”, “Gut microbiota”, “Microbes”, “Microorganisms”, “Age”, Exposure”, “Contaminants”, “Dysbiosis”, “Diet”, “Lifespan”)

| **Table S1**. Recompilation of different scientific articles about microbiota related experiments with *Drosophila melanogaster* classified depending on the age of the model at the start of the experiment. (With this information, Fig S1 was constructed) | | | | | |
| --- | --- | --- | --- | --- | --- |
| **1 or less** | **2-5 days** | **6-10 days** | **10-20 days** | **>20 days** | **Not disclosed** |
| Gacia-Lozano et al, 2020 | Henry et al, 2024 | Jans et al, 2024 | Leech et al, 2021 | Saadjad et al, 2024 | Gayathri et al, 2024 |
| Wang et al, 2024 | Fujita et al, 2024 | Arias-Rojas and Iatsenko, 2022 | Beghelli et al, 2024 | Akiki et al, 2024 | Lannwarne and Dobson, 2025 |
| Bombin et al, 2020 | Cruz et al, 2024 | Mazzucco and Schötterer, 2021 | Go et al, 2024 | Grenier et al, 2020 | Ramírez-Camejo etal, 2017 |
| Shrivastava et al, 2023 | Schaffer et al, 2024 | Yu et al, 2020 | Singh et al, 2024 |  | Trinde et al, 2017 |
| Ma et al, 2021 | Bost et al, 2018 | Westfall et al, 2018 | Ma et al, 2021 |  | Lee et al, 2017 |
| García-Roa et al, 2022 | Yixin et al, 2017 | Cao et al, 2023 | Daisley et al, 2018 |  | Stuivenberg et al, 2022 |
| Dong et al, 2022 | Qiao et al,2019 | Capo et al, 2019 | Tan et al, 2020 |  | Lee et al, 2023 |
|  | Henry et al, 2020 | Mohadam et al, 2018 | Lee et al, 2022 |  | Chifiriuc et al, 2022 |
|  | Ignatiou et al, 2024 | Troha et al, 2019 | Ankrah et al, 2021 |  |  |
|  | Lee et al, 2022 | Chandler et al, 2022 |  |  |  |
|  | Kosakamoto et al, 2020 | Yau et al, 2025 |  |  |  |
|  |  | Dodge et al, 2023 |  |  |  |
